# Effect of Nanosilica and Bentonite as Mycotoxins Adsorbent Agent in Broiler Chickens’ Diet on Growth Performance and Hepatic Histopathology

**DOI:** 10.3390/ani11072129

**Published:** 2021-07-17

**Authors:** Abdallah A. Ghazalah, Mamduh O. Abd-Elsamee, Kout Elkloub M. E. Moustafa, Mohamed Abdelrazik Khattab, Abd-Elrahim A. A. Rehan

**Affiliations:** 1 Department of Animal Production, Faculty of Agriculture, Cairo University, Giza 12613, Egypt; ghazalah@gmail.com (A.A.G.); mamdouh20466@yahoo.com (M.O.A.-E.); 2 Agriculture Research Center, Institute of Animal Production Research, Ministry of Agriculture, Giza 12619, Egypt; dr.koutelkloub@yahoo.com; 3 Department of Cytology and Histology, Faculty of Veterinary Medicine, Cairo University, Giza 12613, Egypt; mabdelrazik@cu.edu.eg

**Keywords:** broiler, growth, histopathology, mycotoxins, Nano-Silica

## Abstract

**Simple Summary:**

Mycotoxins cause significant economic losses in feed ingredients, nutritional value, feed palatability, and the poultry industry. Thus, there is a need for ways to eradicate or inactivate mycotoxins in chicken feed. The present feeding trial aims to evaluate the use of nanosilica and bentonite to prevent the harmful effects of a mycotoxin-contaminated diet on broiler performance, histopathological, and carcass traits. The obtained results revealed significant improvements in broiler growth performance resulting from the addition of nanosilica at 0.20% and bentonite at 0.50%. Additionally, the hepatoprotective efficacy of nanosilica was evident at different dose levels. Consequentially, it could be used in broiler’s contaminated diets without negatively affecting birds’ health.

**Abstract:**

Mycotoxins are toxic secondary metabolites produced by different strains of fungi, such as aspergillus, fusarium, and penicillium that can contaminate feed ingredients or the entire feed of poultry and animals. Mycotoxins can cause many serious complications to both humans and animals due to carcinogenic, mutagenic, and immunosuppressive disorders. Therefore, the present experiment aims to investigate the effect of broiler chickens’ diets supplemented with different levels of nanosilica (NS) as an adsorbent agent of mycotoxins on their growth performance and hepatic histopathology. Detectable levels of toxins were present in the feed before feeding, and all levels of mycotoxins were above the normal limit. A total of 180 one-day-old male Arbor Acres broiler chickens were allocated randomly to six treatment groups with three replicates per group, including ten chickens per replicate. The experiment lasted for five weeks, and dietary treatments included control diet and diets with four levels of nanosilica as 0.05%, 0.10%, 0.15%, and 0.20% as well as 0.50% bentonite (fixfin^®^ Dry) diet. Bodyweight, body weight gain, average daily feed intake, and feed conversion ratio were measured weekly. At the end of the fifth week, six chickens per treatment were sacrificed to investigate the effects of NS and bentonite on carcass characteristics and hepatic histopathology. The results showed that providing broiler chickens’ diets with an adsorbent agent, such as NS or bentonite, can reduce the side effects of mycotoxins and enhance their growth performance. The best record was achieved with NS at 0.20%, compared with the control group and other dietary treatment groups. Accordingly, 0.20% of NS could be used in broiler chickens’ diets to minimize the harmful effects of mycotoxins.

## 1. Introduction

Mycotoxins are toxic secondary compounds synthesized under specific conditions by certain fungal species that can be present on field crops during harvest, storage, processing, or feeding. The most common mycotoxins are Aflatoxins (AF), Ochratoxin A (OTA), Citrinin, patulin, trichothecenes, Deoxynivalenol (DON), T2 toxin (T2), fumonisins, and Zearalenone (ZEN) [1]. More than 500 mycotoxins could be considered toxigenic. It is estimated that 25–50% of grains produced around the world are contaminated with mycotoxins to varying degrees, with 5–10% of them being irreversible, resulting in major economic losses [2,3]. Mycotoxins are considered one of the most serious and dangerous problems affecting poultry and animals, and consequently, affect public health and productivity rates. Therefore, removing mycotoxins from poultry feed is becoming an intractable problem. Currently, there are different methods used to alleviate the side effects of mycotoxins, especially natural sources, such as nanosilica (NS) from rice husk and clay minerals which have been considered as promising adsorbents for highly efficient removal of toxic mycotoxins from animal feeds [4]. The presence of mycotoxins in animal feed may destroy or reduce the nutritional value and palatability of feeds; thus, it makes the animals refuse to eat, grow slower, fall ill easily, and even die [5]. The main problem related to mycotoxin-contaminated animal feed is carcinogenic diseases. Carcinogenicity of some mycotoxins occurs when the toxicity passes unaltered through metabolic processes and accumulates in the tissues, causing an array of metabolic disturbances and, consequently, poor animal productivity [1]. Ducks are the most susceptible to mycotoxicosis in poultry, followed by turkeys, quails, broilers, and layers. Symptoms include fatty livers, kidney diseases, leg and bone deformities, decreased weight gain and productivity, immunosuppression, small and poor-quality eggs, and pigmentation issues [6].

For the detoxification and decontamination of feed with mycotoxins, different physical, chemical, and biological methods are used, including eliminating mycotoxins from contaminated feed ingredients, decreasing the bioavailability of such mycotoxins in the gastrointestinal tracts of animals, or directly degrading mycotoxins in feed.

Accordingly, some suggested approaches include using adsorbent agents, chemical treatment, and several microbial species that have been identified for their ability to biotransform mycotoxins into less toxic forms [7].

Many types of mycotoxin binders are used as a feed supplement to alleviate toxicity in the feed. Bentonites and zeolites are commonly used as feed additives due to their good cation exchange reactions and opposite polarity [8,9]. However, the abovementioned mycotoxin adsorbents often bind to other minerals and vitamins present in the diet rendering them inactive. Although conventional methods are constantly improving, recent research findings are looking for innovative solutions. Nanotechnology has the potential to provide some solutions since the possible applications of nanoparticle ingredients as feed additives are believed to be more effective to minimize the health effects of mycotoxins. Nanotechnology approaches appear to be promising, compelling, and low-cost ways to reduce the side effect of mycotoxins on animals’ health. Aluminum silicate and other nanoclay types have been introduced as unique additives possessing sizeable surface area, higher porosity, strong cation exchange activities, and more active sites, which interact with mycotoxins [10]. There are two types of nanoparticles, namely natural and synthetic ones [11]. The synthetic nanoparticles are synthesized, either by mechanical grinding, engine exhausts, and smoke, or by physical, chemical, biological, or hybrid methods [12]. Clay minerals are used in poultry nutrition for several reasons, including toxin binding [13], improvement of the enzymatic activity in the small intestine [14], and control of ammonia emission into the environment [15]. In addition, natural clays could change GIT enzymatic secretion by raising the GIT fluids’ pH [16]. In the digestive tract of poultry fed a sepiolite-supplemented diet, increased secretion of digestive enzymes was attributed to a decrease in digesta viscosity [17].

The objectives of the current study were to determine the efficacy of using new strategies to ameliorate the toxic effects of mycotoxins in broiler chickens’ diets supplemented with NS as an adsorbing agent of mycotoxins by assessing the growth performance of broiler chickens and liver hematology.

## 2. Materials and Methods

### 2.1. Ethical Approval

The Institutional Animal Care and Use Committee of Cairo University approved the experimental protocol used in this study with approval reference number CU II F 3 21.

### 2.2. Experimental Chickens, Design, and Management

A total of 180 one-day-old male Arbor Acers broiler chickens of similar body weight were distributed randomly into six treatment groups, with three replicates for each group containing ten chickens. Birds were housed in galvanized wire cage batteries (10 birds per m^2^). Feed was provided ad libitum and freshwater was automatically available all time through stainless steel nipples. Birds were raised in a semi-closed house. Room temperature was controlled and thermostatically regulated by two heaters. During the first week, the room temperature was set at 33 °C, gradually reduced by 3 °C every week until it reached 25 °C. Lighting was available 24 h for the first three days of the trials. Then, the lighting schedule was 18 h of light, 6 h of dark daily during the whole experimental period. In this experiment, birds were vaccinated against avian flu, New-Castle disease, and IB at seven days of age. On the 18th day of age, birds were revaccinated against the New-Castle disease. The trial was carried out at the Poultry Research and Training Unit, Faculty of Agriculture, Cairo University, Giza, Egypt. The trial was carried out during April and May 2019.

### 2.3. Materials of Histological Examination

Samples were taken from three birds per treatment. Dissected liver samples were trimmed and fixed in ten neutral buffered formalin. Samples were dehydrated and processed in serial grades of ethanol, cleared in xylene, and impregnated by Paraplast tissue-embedding media. Then, five microns thick tissue sections were cut by a rotatory microtome. Sections were fixed to glass slides and stained by standard hematoxylin and eosin stain for general histological examination [18]. Samples were examined and imaged by using a full HD microscopic imaging system (Leica Microsystems GmbH, Wetzlar, Germany). A microscopic examination lesion score system was performed according to [19].

### 2.4. Adsorbing Agent Sources

The dietary sources of silica used in the current study as an adsorbent agent of mycotoxins were commercial nanoclay (fixfin^®^ Dry) or bentonite (from Kemin company, Belgium). The NS was synthesized from rice husk prepared in the Poultry Nutrition Department Lab, Animal Production Research Insinuate, ARC, Egypt [20].

Corn and soybean meal-based diets were formulated according to the nutritional requirement recommended by the strain catalog of Arbor Acers. Basal diets were formulated for starters (1–14 days old), growers (15–28 days old), and finishers (29–35 days) as shown in Table 1; yellow corn is naturally contaminated with mycotoxins “aflatoxins, ochratoxins, and T-2 toxins” detected in the mash feed before feeding, using Charm Mycotoxin EZ-M Reader from NutriAd International, Belgium.

The experiment lasted for five weeks, and dietary treatments for the control group were provided through diets free of feed additives. Other diets with four levels of NS were 0.05%, 0.10%, 0.15%, and 0.20%, and bentonite (fixfin^®^ Dry) represented 0.50% of the feed diet. For different levels of NS, it was mixed with soybean as a carrier, then added to the diet.

### 2.5. Body Weight, Body Weight Gain, Feed Intake, Feed Conversion Ratio, and Slaughter Traits

Body weights were recorded in all replicates individually at the end of each week. Weights were, therefore, used to calculate the mean body weight. Feed intake was assessed weekly as follows: feed intake = (feed offered−feed residual)/number of birds per replicate. Feed Conversion Ratio (FCR) was calculated as feed intake (g)/weight gain (g). Mortality was also recorded throughout the trial period. Six chickens per treatment were sacrificed at the end of the fifth week to explore treatment effects on carcass characteristics and hepatic histopathology. The chickens were weighed and slaughtered. Then, the heart, spleen, liver, kidney, bursa of Fabricius, body fat, and gizzard were also weighed. The carcass and organs were estimated as the percentage of live body weight.

### 2.6. Statistical Analysis

The data collected were statistically analyzed by the least-squares procedure of the General Linear Model (GLM) of the Statistical Analysis System program [21] to study the effect of NS on broiler chickens’ performance. To investigate the significance of the means, Duncan’s New Multiple Range Test [22] was used and *p*-values less than 0.05 were considered statistically significant.

The model used in the analysis was

Y_ij_: µ + T_i_ + ε_ij_

where Y_ij_ is the value of the respective variable, µ refers to the overall mean of the respective variable, T_i_ signifies the effect caused by the ijth Nano-Silica, i: 1 to 6 (1: control, 2: NS 0.05%, 3: NS 0.10%, 4: NS 0.15%, 5: NS 0.20%, and bentonite 0.50%). ij stands for a random error associated with the ijth observation and is assumed to be independently and normally distributed.

## 3. Results

### 3.1. Detection of Dietary Mycotoxins

The control diets used in the current study were mainly detectable in different mycotoxins. Table 2 indicates the levels of mycotoxins in different stages of feeding.

### 3.2. Growth Performance

According to the results presented in Table 3, the BW of chickens fed diets supplemented with 0.20% NS showed a significant change compared with other treatment groups at the different stages of growing chickens at 14, 21, 28, and 35 days. The highest BW was recorded for NS at 0.20% compared with other treatments, followed by NS at 0.15%.

Table 3 shows that all NS treatments achieved higher values of BWG, compared with the control group, at the period of 7 to 14 days. The highest BWG was achieved with NS at 0.20%. Additionally, at the period of 22–28 days, the supplementation of 0.20% NS recorded the highest BWG. On the other hand, the addition of bentonite recorded the highest BWG at the period of 29–35 days compared with different treatments. Generally, at the overall period, the best BWG was obtained using 0.20% NS followed by 0.15% NS treatment.

Broiler chickens fed the diet with NS at 0.15% and 0.20% showed a significant increase in FI during 7–14 days of age compared with other treatments (Table 3). The diet with NS at 0.15% and 0.05% resembled the control’s results and significantly higher than other treatments during 15–21 days. On the other hand, the addition of bentonite at 0.50 recorded the highest FI during 22–28 days. Feed intake was significantly increased with the addition of bentonite 0.50% during 29–35 days compared to all the treatments. Regarding the overall FI (7–35 days), the addition of 0.15% of NS and 0.50% of bentonite showed a significant increase in FI compared with other treatments and the control group.

Statistical analysis indicated that NS at levels of 0.10% and 0.20% had a significant effect compared with all treatments on FCR during 7–14 days of age. Chickens receiving NS at 0.20% recorded a significant improvement of FCR compared with the control and other treatment groups during 15–21 days and 22–28 days of age. The best FCR value was recorded with treatment supplemented with commercial nanoclay (bentonite 0.50%) followed by those having 0.05% and 0.10% of NS, compared with the control group at the period of 29–35 days of age. Considering the overall FCR, the addition of NS or bentonite improved FCR, and the best FCR was achieved with NS at 0.20%, compared with other treatments (*p* < 0.05).

### 3.3. Carcass Characteristics

The data presented in Table 4 shows that no significant effects of dietary treatments on carcasses, abdominal fat, bursa, thymus, and relative organs weight (liver, heart, gizzard, and spleen) were observed.

### 3.4. Histological Findings

**Figure 1:**
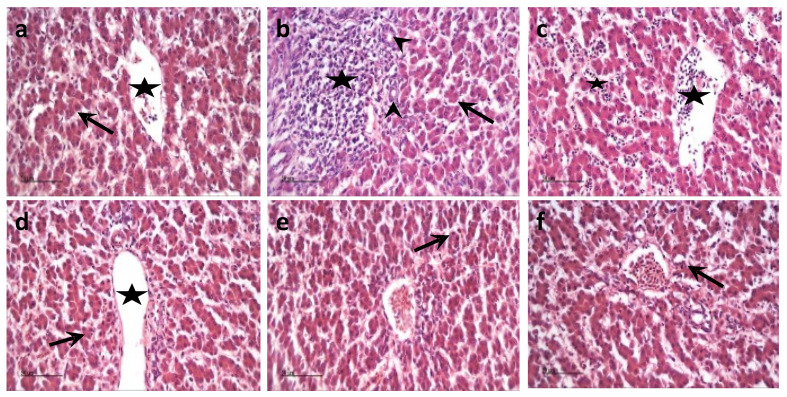
The hepatoprotective efficacy of nanosilica in different doses: (**a**) control normal group hepatic samples with normal morphological features of the hepatic parenchyma, (**b**) hepatic samples of nanosilica in the 0.05% administrated group, (**c**) hepatic samples of nanosilica in the 0.10% administrated group, (**d**) hepatic samples of nanosilica in the 0.15% administrated group, (**e**) hepatic samples of nanosilica in the 0.20% administrated group, and (**f**) hepatic samples of nano-bentonite administrated group. H&E stain. X400. Scale bar: 50 microns.

Microscopic examination of different liver tissue sections from different groups revealed that the normal histomorphological structures of hepatic tissue were demonstrated in control samples (Figure 1a), including normally appearing blood vessels (star), portal areas, and radiating plates of intact hepatocytes with vesicular centrally situated nuclei (arrow). Mild degenerative changes were recorded in hepatocytes with dark pyknotic nuclei (arrow). Variable protective effects were demonstrated in different treated groups on morphological structures. However, the best records were found in nanosilica 0.20% (Figure 1e), nano-bentonite (Figure 1f), and nanosilica 0.15% (Figure 1d), respectively, with few scattered degenerated hepatocytes (arrows) and normal blood vessels as well as sinusoids—except mild dilatation of hepatic blood vessels—in NS 0.15% samples (star, Figure 1d). Examined hepatic tissue sections of NS 0.05% showed the existence of focal periportal inflammatory cell infiltrations (star) with the proliferation of bile ducts (arrowhead), and few degenerated hepatocytes (arrow) (Figure 1b). Moreover, NS 0.10% samples demonstrated marked dilatation and congestion of hepatic sinusoids (star) with minimal inflammatory cell infiltrates (Figure 1c). Number of lesion score of liver has been shown in Table 5.

## 4. Discussion

The European Commission [23] published maximum levels of several mycotoxins in the European Union (EU) as aflatoxin 20 ppb/kg of feed, ochratoxin 100 ppb/kg of feed, T2 toxins 250 ppb/kg of feed, and fumonisins 20 mg/kg of feed. All levels of mycotoxins in control diets used herein (Table 2) were above the maximum recommended level of the European Guidance values except the T2 toxins, and the highest toxin level was fumonisins, which promotes the use of different sources of silica as an adsorbent agent in broiler chickens’ feed. It is well known that intoxication is caused by mycotoxins, and it is one of the most serious risks, especially for poultry. Moreover, quality deterioration and decrease in the commercial value of feed ingredients are all possible negative effects of their bioaccumulation. So, the use of anti-mycotoxin agents, particularly in the form of nanoparticles, may improve the growth rate and digestibility.

The enhancement of performance may be attributed to the ability of NS and bentonite to adsorb mycotoxins. Consequently, they improved feed utilization. Numerous studies reported that clay minerals bentonite in animal feed induces the decrease of the harmful impacts of mycotoxins in vivo [24]. Formerly, “Ref. [25]” reported that supplementation of diets with two types of bentonites (clinoptilolite and zeolite) at 1% each as potential binders of three mycotoxins, aflatoxin, ochratoxin, and T2 toxin, present in broilers’ diets (at levels not exceeding the EU maximum) exerted more prominent effects on the growth performance and GIT function of broiler chickens. Similarly, dietary nano hydrated magnesium aluminum silicate supplementation resulted in more pronounced changes in broiler chicken growth and digestive function than the traditional hydrated magnesium aluminum silicate equivalent [26]. These results may be attributed to the beneficial properties of nanosilica: high adsorbent capacity, the high surface area of layers, and the charge which binds different mycotoxins. In the present study, mycotoxins in the studied feed were overcome by nanosilica adsorbents with unique plate structures, large specific surface areas, excellent stability, and a wide range of functionalities, which could be used as multi-mycotoxin binders to mitigate the negative effects on broiler chickens’ diets. The capacity of nanosilica to adsorb mycotoxins may be responsible for the increased BWG. As a result, feed consumption and body weight increased. “Ref. [7]” investigated diets containing hydrated sodium calcium aluminum silicate at a level of 0.30% as one of the physical detoxifiers that increased the average daily gain of broiler chickens. “Ref. [27]” reported that the greatest weight gain was observed by supplementing nanoclay to turkey feed. Furthermore, “Ref. [28]” suggested that sodium silicate improved the average daily gain of broiler chickens at a level of 0.50%. The same trend was obtained by [29] who reported that the inclusion of silicate minerals in the diets of broiler chickens improved the weight gain, and they explained that this improvement may be due to the action of silicate minerals. As a result, increasing the digestibility of some nutrients while lowering the GIT passage rate lengthens the time nutrients are digested.

In this respect, “Ref. [30]” reported that the FI of a treated group with a local mycotoxin binder was significantly higher than that of the control group, leading to improved performance. “Ref. [31]” found that hydrated sodium calcium aluminosilicates in broiler diets ameliorate the decreased feed intake and increased weight gain at 2 mg/kg of AFB1, whereas 0.2% SO partially recovered the impaired growth performance. The improvement of FCR may be due to the improvement of the enzymatic activity of GIT by raising fluids’ pH and decreasing the viscosity of digesta in the digestive tract of poultry. The findings of the present study agree with those of [27], who reported that the addition of 0.50% nanoclay as an adsorbent agent of mycotoxins in the diets of turkey improved FCR compared with the control group. Furthermore, “Ref. [32]” recorded that FCR was significantly improved with the addition of sodium bentonite at 0.5%. Similarly, “Ref. [33]” incorporated zeolite with a nanostructure at 0.25% and 1% in the broiler chickens’ diet contaminated with 500 ppb aflatoxin and reported a reduction of toxic effects of aflatoxin on the growth performance.

The present result agreed well with that of [34], who discovered that the experimental therapies, which included two forms of bentonites in broiler chicken diets, had no statistically significant differences in carcass yield and relative weight of heart, spleen, liver, bursa of Fabricius, and gizzard. However, “Ref. [35]” found that supplemented sodium bentonite and coumarin to the aflatoxin-contaminated rabbits’ diet improved the carcass characteristics and live body weight.

Mycotoxins can exert histopathological changes that can interfere with broiler growth rate. Therefore, the addition of nanosilica and bentonite showed protective effects on the histopathological changes in liver sections, leading to the enhancement of enzyme secretion and improved performance. Agreeing with this study, “Ref. [36]” concluded that the addition of silica to diets containing AFB1 significantly reduced the incidence and severity of the hepatic histopathology changes associated with aflatoxicosis and the amount of AFB1 residue in the liver and improved performance. In another study, “Ref. [31]” proved showed that treatment by Hydrated Sodium Calcium Aluminosilicates (HSCAS) in broiler diets ameliorated the negative effects of AFB on growth performance and liver damage. Accordingly, Ref. [37] showed that the addition of bentonite was more effective in preventing histopathological effects from aflatoxicosis, compared with activated charcoal or fuller’s earth. However, “Ref. [32]” revealed that no mycotoxins were detected in the examined liver and breast muscles when two types of bentonites in broiler diets were used. Additionally, “Ref. [35]” investigated that no histopathological changes were observed in the liver tissues of rabbits fed with aflatoxin-contaminated diets containing sodium bentonite and coumarin. Furthermore, the current study agreed with [30], who found that the addition of local mycotoxin binder to broiler diets showed a protective effect on liver tissue and improved performance. “Ref. [38]” reported that the addition of bentonite to the contaminated broiler diets with aflatoxin and fumonisin showed a reduction in the multifocal and varied cytoplasmatic vacuolization through the reduction of the incidence and severity of the hepatic histopathology changes associated with aflatoxicosis.

## 5. Conclusions

This study showed that the contaminations of mycotoxins in feed can affect the growth rate of broilers. It is necessary to use adsorbent agents, such as bentonite or silicate and especially in the form of nanosilica, which can be more active to minimize the harmful effects of mycotoxins. Moreover, the study addresses the prevention and control strategies of mycotoxins using clay minerals in the form of nanosilica and bentonite. It could be concluded that the addition of nanosilica and bentonite improved the performance and the health of broiler chickens at 0.20% and 0.50% levels, respectively. The findings suggest that there is a need to conduct more research in the field using nanomaterials as adsorbent agents for mycotoxins. The obtained results can be implemented in different areas, including the utilization of rice husks as a source of nanosilica acting as an adsorbent agent of mycotoxins to improve the performance of broiler chickens.

## Figures and Tables

**Table 1:** Composition and calculated analysis of the experimental diets for Arbor Acers broiler during starter (1–14 days old), grower (15–28 days old), and finisher intervals (29–35 days old) ^1^.

Ingredients	Starter	Grower	Finisher
	(1–14 Days)	(15–28 Days)	(29–35 Days)
Yellow corn	51.90	57.31	62.25
Soybean meal 44%	34.99	29.60	24.25
Corn gluten meal 60%	5.00	5.00	5.00
Soybean oil	3.28	3.82	4.45
Limestone	1.85	1.50	1.40
Mono calcium phosphate	1.45	1.30	1.20
Vitamins and minerals premix ^2^	0.30	0.30	0.30
DL-methionine	0.28	0.24	0.22
L-lysine hydrochloride	0.31	0.30	0.32
L-threonine	0.09	0.08	0.06
Sodium chloride	0.35	0.35	0.35
Choline chloride 60%	0.10	0.10	0.10
Sodium bicarbonate	0.10	0.10	0.10
Total %	100%	100%	100%
Calculated Composition ^3^			
Crude protein (%)	23.00	21.00	19.00
Metabolizable energy (Kcal/kg)	3000	3100	3200
Crude fiber (%)	3.75	3.48	3.20
Crude fat (%)	5.65	6.35	7.13
Calcium (%)	1.00	0.87	0.80
Available phosphorus (%)	0.48	0.44	0.40
Lysine (%)	1.44	1.29	1.16
Methionine (%)	0.50	0.51	0.47
Methionine + cystine (%)	1.08	0.99	0.91
Threonine (%)	0.97	0.88	0.78
Sodium (%)	0.20	0.20	0.20

^1^ According to the nutritional requirement recommended by the strain catalog of Arbor Acers. ^2^ Vitamins, for every 1.5 kg, contains vitamins (Vit). A 12,000,000 IU, Vit. D_3_ 4000,000 IU, Vit. E 100,000 mg, Vit. K_3_ 3000 mg, Vit. B_1_ 2500 mg, Vit. B_2_ 5000 mg, Vit. B_6_ 5000 mg, Vit. B_12_ 20 mg, nicotinic acid 55,000 mg, pantothenic acid 15,000 mg, folic acid 2000 mg, biotin 200 mg, choline 400,000 mg, and carrier calcium carbonate up to 1.5 kg. Minerals, for every 1.5 kg, contains iron 80,000 mg, copper 8000 mg, zinc 100,000 mg, manganese 120,000 mg, iodine 1000 mg, selenium 300 mg, cobalt 200 mg, and carrier calcium carbonate up to 1.5 kg. ^3^ According to the nutritional requirement recommended by the strain catalog of Arbor Acers.

**Table 2:** Mycotoxin concentration determined in the diets fed in the different stages of the trials.

Dietary Group	AFB1 (ppb/kg)	OA (ppb/kg)	T2 (ppb/kg)	FB (mg/kg)
Starter	35.33	120	74	120
Grower	37.25	173	88	135
Finisher	39.22	190	92	220

AFB1: Aflatoxin B1. OA: Ochratoxin. T2: T2 Toxins. FB: Fumonisins.

**Table 3:** Effects of nanosilica and bentonite supplementation on the performances of male broiler chickens between 0 and 35 days of age.

Items	Nanosilica Levels							
	Control	0.05%	0.10%	0.15%	0.20%	Bentonite 0.50%	SEM	*p*-Values
	Body Weight (BW), g/bird							
7 d	175.7	175.7	175.7	175.7	175.7	175.7	0.08	ns
14 d	419.3 ^d^	429.3 ^c^	441.3 ^b^	441.7 ^b^	451.3 ^a^	416.0 ^d^	1.50	<0.050
21 d	844.0 ^c^	873.3 ^b^	874.0 ^b^	878.0 ^b^	896.0 ^a^	848.7 ^c^	2.94	<0.050
28 d	1397.7 ^c^	1395.3 ^c^	1398.3 ^c^	1465.0 ^b^	1497.3 ^a^	1394.0 ^c^	3.39	<0.050
35 d	1925.0 ^d^	1977.0 ^c^	1975.0 ^c^	2032.3 ^b^	2061.0 ^a^	2013.7 ^b^	9.02	<0.050
	Body Weight Gain (BWG), g/bird							
7–14 d	244.3 ^d^	253.7 ^c^	265.7 ^b^	266.0 ^b^	275.7 ^a^	240.3 ^d^	1.54	<0.050
15–21 d	424.7 ^c^	444.3 ^a^	432.7 ^b^	436.3 ^b^	445.0 ^a^	432.7 ^b^	2.45	<0.050
22–28 d	554.0 ^c^	522.3 ^e^	524.3 ^e^	587.3 ^b^	601.3 ^a^	545.7 ^d^	2.84	<0.050
29–35 d	527.3 ^c^	581.7 ^b^	576.7 ^b^	567.3 ^b^	563.7 ^b^	619.7 ^a^	5.98	<0.050
7–35 d	1750.0 ^d^	1801.3 ^c^	1799.3 ^c^	1856.7 ^b^	1885.3 ^a^	1838.0 ^b^	9.03	<0.050
	Feed Intake (FI), g/bird							
7–14 d	281.0 ^d^	288.0 ^c^	282.7 ^cd^	305.3 ^a^	295.3 ^b^	277.0 ^d^	1.93	<0.050
15–21 d	570.3 ^a^	572.3 ^a^	567.3 ^b^	573.0 ^a^	566.7 ^bc^	564.3 ^c^	0.99	<0.050
22–28 d	832.0 ^d^	844.3 ^a^	838.0 ^c^	840.7 ^b^	843.3 ^a^	845.3 ^a^	0.73	<0.050
29–35 d	1092.7 ^b^	1069.0 ^c^	1062.7 ^d^	1070.7 ^c^	1056.0 ^e^	1106.7 ^a^	1.36	<0.050
7–35 d	2775.3 ^b^	2772.7 ^b^	2750.0 ^d^	2789.3 ^a^	2761.0 ^c^	2792.3 ^a^	3.19	<0.050
	Feed Conversion Ratio (FCR)							
7–14 d	1.150 ^a^	1.136 ^a^	1.066 ^b^	1.146 ^a^	1.073 ^b^	1.153 ^a^	0.009	<0.050
15–21 d	1.356 ^a^	1.290 ^c^	1.316 ^b^	1.313 ^b^	1.273 ^d^	1.306 ^bc^	0.007	<0.050
22–28 d	1.500 ^c^	1.620 ^a^	1.603 ^a^	1.430 ^d^	1.403 ^e^	1.550 ^b^	0.008	<0.050
29–35 d	2.083 ^a^	1.840 ^bc^	1.840 ^bc^	1.896 ^b^	1.886 ^b^	1.795 ^c^	0.020	<0.050
7–35 d	1.590 ^a^	1.540 ^b^	1.530 ^b^	1.503 ^c^	1.466 ^d^	1.520 ^bc^	0.008	<0.050

^a–e^ Means within a row sharing different superscripts differ significantly (*p* < 0.05). SEM = Standard Error of Mean, BW= Body Weight, BWG = Body Weight Gain, FI = Feed Intake, FCR = Feed Conversion Ratio, and ns = Nonsignificant.

**Table 4:** Effects of nanosilica and bentonite supplementation on eviscerated carcass and some internal organs of male broiler chickens at 35 days of age * (expressed as % of body weight) *.

Treatments	Carcass (%)	Liver (%)	Heart (%)	Gizzard (%)	Spleen (%)	Abd. Fat (%)	Bursa (%)	Thymus (%)
Control	73.00	2.30	0.47	1.95	0.11	0.45	0.21	0.37
NS 0.05%	73.33	2.31	0.48	2.17	0.14	0.42	0.22	0.30
NS 0.10%	74.95	2.41	0.50	2.11	0.12	0.59	0.23	0.35
NS 0.15%	72.47	2.25	0.60	2.17	0.14	0.67	0.22	0.39
NS 0.20%	73.10	2.36	0.60	2.21	0.12	0.56	0.18	0.29
Bentonite 0.50%	74.14	2.11	0.62	1.93	0.13	0.46	0.24	0.29
SEM	2.50	0.41	0.05	0.25	0.03	0.09	0.01	0.03
*p*-Values	ns *	ns	ns	ns	ns	ns	ns	ns

* The percentage of eviscerated carcass and some internal organs to live body weight. NS = Nanosilica, SEM = Standard Error Means, Abd. fat = Abdominal fat, and ns * = Nonsignificant. Values in each column are means of three replicates (ten birds each) for each treatment (n = 6).

**Table 5:** Efficacy of nanosilica and bentonite in the amelioration of the toxic effects of mycotoxins on liver lesion score.

Treatments	Degenerative Changes of Hepatocytes	Inflammatory CellsInfiltration	Hepatic Vasculatures/ Sinusoidal Diltation	Proliferation of Bile Ducts
Control	+	-	-	-
NS 0.05%	+	++	-	++
NS 0.10%	+	+	+++	-
NS 0.15%	+	-	+	-
NS 0.20%	+	-	-	-
Bentonite 0.50%	+	-	-	-

Recorded lesions were graded and scored according to [19]. Where (-) is nil, (+) mild, less than 15% of examined samples. (++) moderate (16–35% of examined samples), (+++) severe (more than 35% of the examined samples).

## Data Availability

Data are available if requested.
